# Serum AXL is a potential molecular marker for predicting COVID-19 progression

**DOI:** 10.3389/fimmu.2024.1394429

**Published:** 2024-05-10

**Authors:** Jianbin You, Rong Huang, Ruifang Zhong, Jing Shen, Shuhang Huang, Jinhua Chen, Falin Chen, Yanli Kang, Liangyuan Chen

**Affiliations:** ^1^ Department of Clinical Laboratory, Shengli Clinical Medical College of Fujian Medical University, Fuzhou, Fujian, China; ^2^ Department of Clinical Laboratory, Fujian Provincial Hospital, Fuzhou, Fujian, China; ^3^ The School of Basic Medical Sciences, Fujian Medical University, Fuzhou, Fujian, China

**Keywords:** AXL, ACE2, SARS-CoV-2 IgG/IgM antibodies, COVID-19, biomarker

## Abstract

**Background:**

The severity, symptoms, and outcome of COVID-19 is thought to be closely linked to how the virus enters host cells. This process involves the key roles of angiotensin-converting enzyme 2 (ACE2) and the Tyrosine protein kinase receptor UFO (AXL) receptors. However, there is limited research on the circulating levels of ACE2 and AXL and their implications in COVID-19.

**Methods:**

A control group of 71 uninfected individuals was also included in the study. According to the Guidance for Corona Virus Disease 2019 (10th edition), a cohort of 358 COVID-19 patients were categorized into non-severe and severe cases. Serum ACE2/AXL levels in COVID-19 patients were detected by enzyme-linked immunosorbent assay (ELISA) at different time points post-COVID-19 infection, including days 0-7, 8-15, 31-179 and >180 days. Serum SARS-CoV-2 IgG/IgM antibodies in COVID-19 patients at the same intervals were assessed by using an iFlash 3000 Chemiluminescence Immunoassay Analyzer. The receiver operating characteristic (ROC) curves were used to assess the diagnostic value of the biological markers, and the association between laboratory parameters and illness progression were explored.

**Results:**

Compared with the uninfected group, the levels of ACE2 and AXL in the COVID-19 group were decreased, and the SARS-COV-2 IgG level was increased. AXL (AUC = 0.774) demonstrated a stronger predictive ability for COVID-19 than ACE2. In the first week after infection, only the level of AXL was statistically different between severe group and non-severe group. After first week, the levels of ACE2 and AXL were different in two groups. Moreover, in severe COVID-19 cases, the serum ACE2, AXL, and SARS-COV-2 IgM levels reached a peak during days 8–15 before declining, whereas serum SARS-COV-2 IgG levels continued to rise, reaching a peak at day 31-180 days before decreasing. In addition, the AXL level continued to decrease and the SARS-COV-2 IgG level continued to increase in the infected group after 180 days compared to the uninfected group.

**Conclusions:**

The levels of serum ACE2 and AXL correlate with COVID-19 severity. However, AXL can also provide early warning of clinical deterioration in the first week after infection. AXL appears to be a superior potential molecular marker for predicting COVID-19 progression.

## 1 Introduction

The COVID-19 pandemic, resulting from SARS-CoV-2 infection, has imposed a significant global burden since its initial report in December 2019 (1, 2). The COVID-19 vaccination programs have been implemented worldwide, and have effectively prevented numerous deaths from SARS-CoV-2 infection (2). However, the rise of persistent viral immune evasion has led to waves of new SARS-CoV-2 variants, such as the Delta and Omicron strains. They are more deadly or contagious, which make existing vaccines less effective (3). The clinical spectrum of the disease ranges from asymptomatic or mild cases to severe manifestations like acute hepatitis or liver failure (4). A substantial portion of individuals with COVID-19 experience severe illness and require intensive care, particularly in the elder (5).

The severity, symptoms, and outcome of COVID-19 is thought to be linked to virus-induced damage to cells and the ability of the virus to evade the host immune system. It has been established that SARS-CoV-2 gains cellular entry by binding to angiotensin-converting enzyme 2 (ACE2) via its spike protein (6, 7). ACE2 expression is detected in various human organs, including the liver, lung, stomach, kidney, and colon, albeit at relatively low levels, especially in the lung (8). Despite sharing the same receptor (ACE2) for cell entry as SARS-CoV, SARS-CoV-2 is more infectious and transmissible. It is plausible that SARS-CoV-2 might rely on additional receptors for effective infection. Tyrosine protein kinase receptor UFO (AXL) functions as a receptor tyrosine kinase transmitting signals from the extracellular matrix to the cytoplasm (9). It has also been identified as a potential receptor for SARS-CoV-2. It interacts directly with the N-terminal domain of SARS-CoV-2 spike glycoprotein to facilitate infection of pulmonary and bronchial epithelial cells (10).

The roles of ACE2 and AXL receptors are crucial in the context of COVID-19. Both of them exist in membrane-bound form and soluble form, and the distinct forms elicit varying impacts (11). Metallo-endopeptidase A and Metalloproteinasesadam are responsible for their hydrolysis, yielding soluble ACE2 and soluble AXL. Researchers have linked elevated levels of soluble AXL to various cancers and have identified it as a cancer biomarker (12). Nonetheless, research on circulating levels of soluble ACE2 and AXL and their implications in COVID-19 patients remains limited.

Our study aimed to analyze the serum levels of ACE2 and AXL in individuals during the acute phase of COVID-19, in comparison to healthy individuals. In addition, we sought to investigate the serum levels of ACE2 and AXL in patients at different time points post-COVID-19 infection and determine if these levels correlate with COVID-19 progression.

## 2 Methods

### 2.1 Study design

Between December 2022 to October 2023, a total of 358 patients with COVID-19 were enrolled in Fujian Provincial hospitals in Fujian Province. COVID-19 was diagnosed through a nasopharyngeal reverse-transcription polymerase chain reaction (RT-PCR) assay or SARS-CoV-2 antigen rapid test. All patients were subdivided into mild or moderate or severe or critical according to the Guidance for Corona Virus Disease 2019 (10th edition) released by the National Health Commission of China (https://www.chinacdc.cn/jkzt/crb/zl/szkb_11803/jszl_11815/202301/t20230107_263257.html). During the study period, effective genomic sequences of COVID-19 cases correspond to the Omicron variant, according to investigation and announcement of the Chinese Center for Disease Control and Prevention (https://www.chinacdc.cn/jkzt/crb/zl/szkb_11803/jszl_13141/).

In our study, patients with mild or moderate COVID-19 were categorized as the non-severe group, and patients with severe or critical COVID-19 were categorized as the severe group. The starting point for all analyses was the day of the positive nucleic acid test or SARS-CoV-2 antigen rapid test (Day 0). Patients were divided into four groups according to different time points: 0-7 days, 8-15 days, 31-179 days, and >180 days. After the patient’s serum is collected, a detailed medical history or telephone return visit must be conducted. If the patient gets another COVID-19 infection, or if the PCR or antigen test taken on the day the serum was collected is positive, the specimen will be excluded.

### 2.2 Participants

A control group of 71 uninfected individuals was also included in the study. The median age of participants was 45 years (IQR 33-62 years), including 28 men and 43 women. Among the 358 cases of patients with COVID-19, 209 non-severe patients and 149 severe patients were included in our study. Severe patients were older than non-severe patients (75 years [IQR 68-83 years] vs. 63 years [IQR 49-75 years]). The study recorded the data for each patient, including age, gender, and comorbidities (e.g. hypertension, diabetes mellitus (DM), chronic cardiovascular disease, chronic pulmonary disease, chronic kidney disease, and tumor). This data were shown in **Table 1**. Informed consent was obtained from all participants, and the experimental protocol was approved by the Institutional Review Board of Fujian Provincial Hospital(Fuzhou, China; No. KY2021-03-013).

**Table 1:** Clinical characteristics of COVID-19 patients and uninfected individuals.

Characteristics	Uninfected(n=71)	Non-severe(n=209)	Severe(n=149)	p-value
Age, y, median(IQR)	45(33~62)	63.00 (49.00~75.00)	75.50(68.00~83.00)	<0.001
Gender, n(%)				<0.001
Male	28(39.4)	118(56.5)	103(69.1)	
Female	43(60.6)	91(43.5)	46(30.9)	
Comorbidities, n(%)				
Hypertension	–	78 (37.3)	102 (68.5)	<0.001
Diabetes	–	40 (19. 1)	56 (37.6)	<0.001
Cancer	–	39 (18.7)	34 (22.8)	0.336
Nephropathy	–	10 (4.8)	17 (11.4)	0.019
Autoimmune disease	–	36 (17.2)	10 (6.7)	0.003

The data conforming to non normal distribution are described as medians and 25th–75th percentile quartile intervals (IQRs) and are compared using the Mann-Whitney U or Kruskal Wallis test. Categorical variables are expressed as numbers and percentages [n (%)], and are compared using contingency table analysis and χ2 tests or Fisher's exact test.

### 2.3 Detection of serum markers

To prepare human serum, blood is drawn from participants, and allowed to clot at room temperature for at least 30 minutes. Then, after centrifugation (8min, 3500 rpm), the supernatant part, the serum, was collected and stored at -80°C.

Serum ACE2/AXL levels were detected through an ELISA assay by using the ACE2/AXL quantitative detection kit (Shanghai Nibei Bio-pharmaceutical Technology Co., Ltd, China). Based on the instructions provided by the kit manufacturer, the ELISA protocol was followed. Briefly, the standards (50 µl; provided by the kit), samples to be tested (10 µl serum samples and 40 µl sample diluent), and blanks were added to a microplate, which precoated with human ACE2/AXL antibody. Then it was incubated at 37°C for 30min. After washing the plate 5 times with phosphate buffer saline (PBS) with Tween, horseradish peroxidase was added to form an immune complex. The unbound substances were washed away, and a substrate solution containing tetramethylbenzidine and urea hydrogen peroxide was added to the microplate. The reaction was stopped by a sulfuric acid solution, and absorbance values were measured by an enzyme-labeled instrument at 450 nm using an enzyme-labelled instrument (Bio-Rad, Hercules, CA, USA). The concentration of ACE2/AXL in serum samples was calculated using its calibration curve. Serum IgM and IgG against SARS-CoV-2 spike 1 protein or nucleocapsid protein were detected by using SARS-CoV-2 antibody reagent kits (YHLO Biotech Co, Ltd, Shenzhen, China) on an iFlash 3000 Chemiluminescence Immunoassay Analyzer (Shenzhen YHLO Biotech Co, Ltd, Shenzhen, China, http://en.szyhlo.com). The kits have catalog numbers C86095M and C86095G for IgM and IgG. The normal reference values were as follows: SARS-COV-2 IgG < 10 AU/ml, SARS-COV-2 IgM < 10 AU/ml.

### 2.4 Statistical analysis

Statistical analyses were performed using SPSS 25.0 software package (SPSS Inc. Chicago, USA) and GraphPad Prism 9.0 (GraphPad Software, USA). Continuous variables were presented as median and interquartile range (IQR). Independent continuous variables were compared using the Mann-Whitney U test or Kruskal-Wallis test, and paired variables with the Wilcoxon matched-pairs signed rank tests or Friedman test. Categorical variables are expressed as numbers and percentages [n (%)], and are compared using contingency table analysis and χ2 tests or Fisher’s exact test. The receiver operating characteristic (ROC) curves were used to assess the diagnostic value of the biological markers, and the association between laboratory parameters and the risk of developing critical disease were explored. A *p*-value < 0.05 was considered statistically significant.

## 3 Results

### 3.1 Expression of ACE2, AXL and SARS-COV-2 IgG/IgM and their diagnostic value for COVID-19

**Figure 1:**
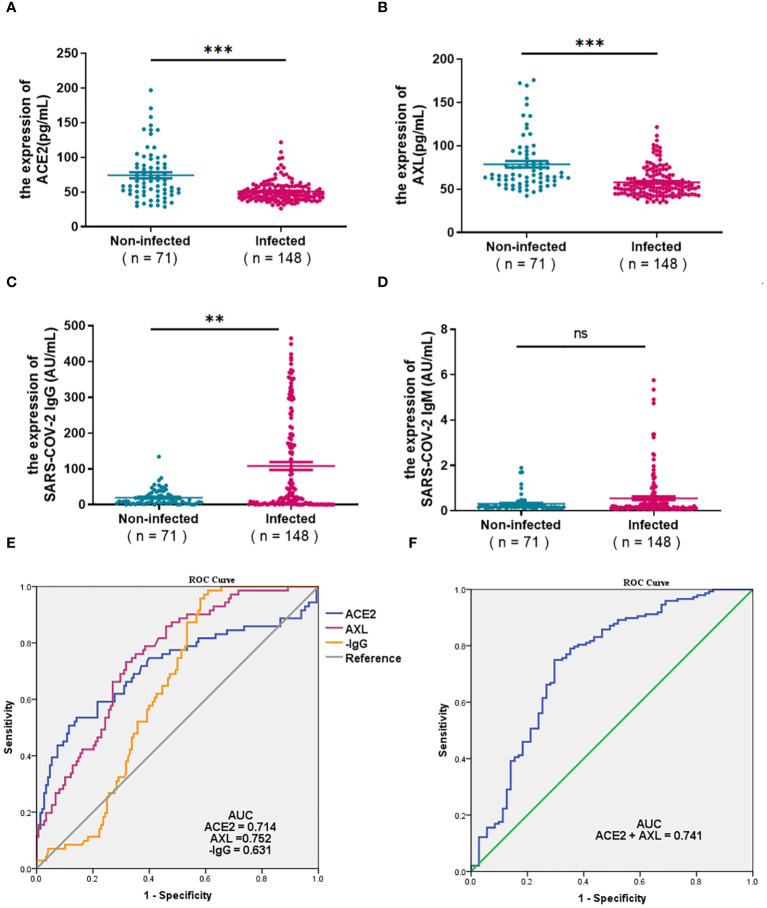
Expression of ACE2, AXL and SARS-COV-2 IgG/IgM and their diagnostic value for COVID-19. The differential expression of serum ACE2, AXL, andSARS-COV-2 IgG/IgM between 71 uninfected individuals and 148 COVID-19 patients with positive nucleic acid in 0-7days were compared withMann Whitney test **(A–D)**. The diagnostic value of ACE2, AXL, minus SARS-COV-2 IgG **(E)** and a combination of ACE2 + AXL **(F)** for COVID-19 wereperformed by ROC curve ( **P <0.01; ***P <0.001; ns, not statistically significant).

The serum ACE2, AXL, and SARS-COV-2 IgG/IgM were compared between a cohort of 71 uninfected individuals and 148 COVID-19 patients with positive nucleic acid in 0-7days. As shown in the **Figures 1A, B**, the levels of ACE2 and AXL in the COVID-19 group were significantly lower than in the non-infected group, while SARS-COV-2 IgG was significantly higher in the COVID-19 group (**Figure 1C**). There was no significant difference in the SARS-COV-2 IgM between the two groups (**Figure 1D**). The ROC curve analysis show that the area under the curve (AUC) of serum ACE2, AXL, and SARS-COV-2 IgG were 0.714, 0.752, and 0.631, respectively (**Figure 1E**). However, the AUC of a combination of ACE2 + AXL (0.741) was not higher than AXL alone, suggesting that AXL had a better ability to predict the risk of COVID-19 (**Figure 1F**).

### 3.2 Expression of serum ACE2, AXL and SARS-COV-2 IgG/IgM in severe and non-severe patients

#### 3.2.1 Days 0-7 after laboratory-confirmed COVID-19

**Figure 2:**
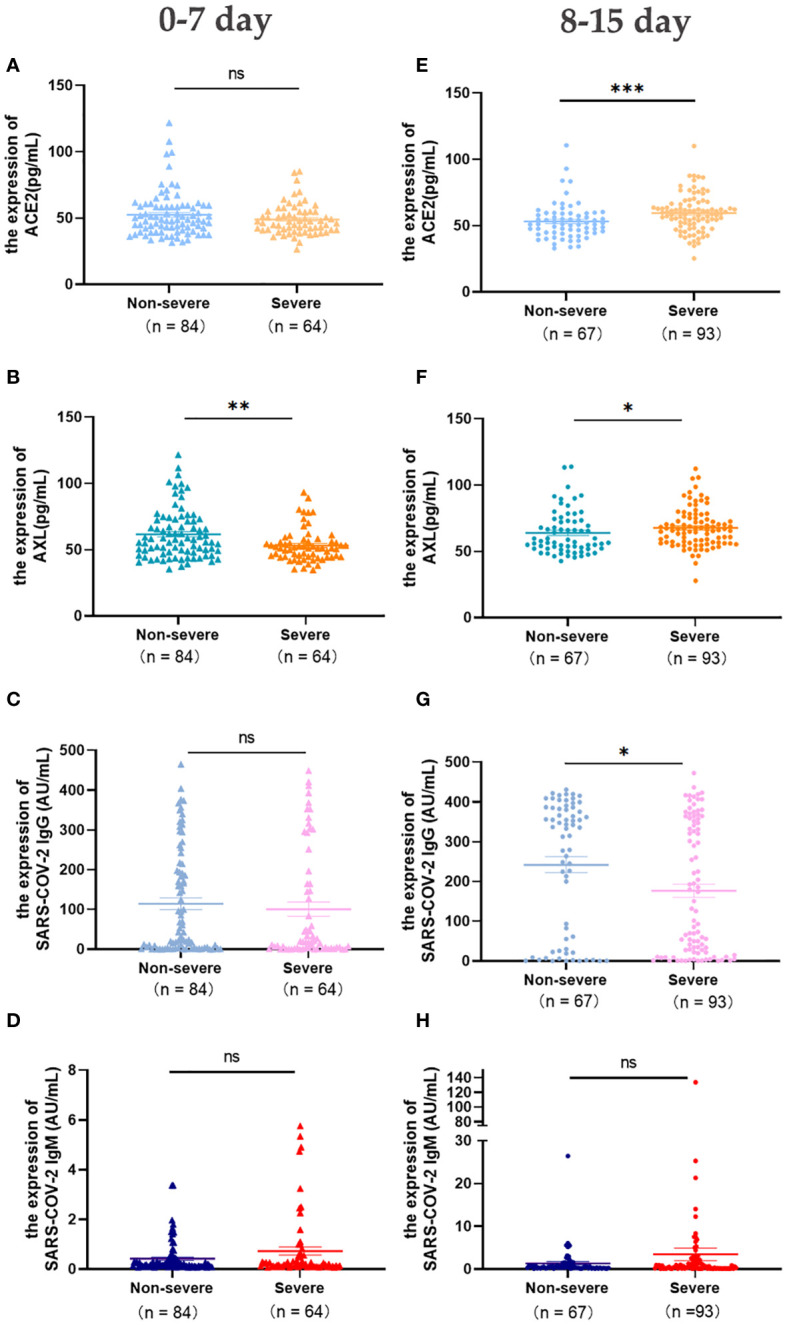
Expression of serum ACE2, AXL and SARS-COV-2 IgG/IgM in severe and non-severe groups at intervals of 0-7 days and 8-15 days were compared with Mann Whitney test **(A-H)** (*P <0.05; **P <0.01; ***P <0.001; ns, not statistically significant).

The levels of ACE2, AXL, SARS-COV-2 IgG/IgM were analyzed in patients with early COVID-19 (first week after laboratory-confirmed COVID-19), including 84 cases of non-severe and 64 cases of severe. The results showed that AXL in the severe group was significantly lower than in the non-severe group, while there were no significant differences in the levels of ACE2, SARS-COV-2 IgG/IgM between the two groups (**Figures 2A-D**).

#### 3.2.2 Days 8-15 after laboratory-confirmed COVID-19

In the second week after laboratory-confirmed COVID-19, the levels of serum ACE2 and AXL in the severe group (n=93) were significantly higher than that in the non-severe group (n=67), while SARS-COV-2 IgG was significantly lower (**Figures 2E-G**). No significant differences were observed in the levels of serum SARS-COV-2 IgM between the two groups (**Figure 2H**).

#### 3.2.3 Days 31-179 and more than 180 days after laboratory-confirmed COVID-19

**Figure 3:**
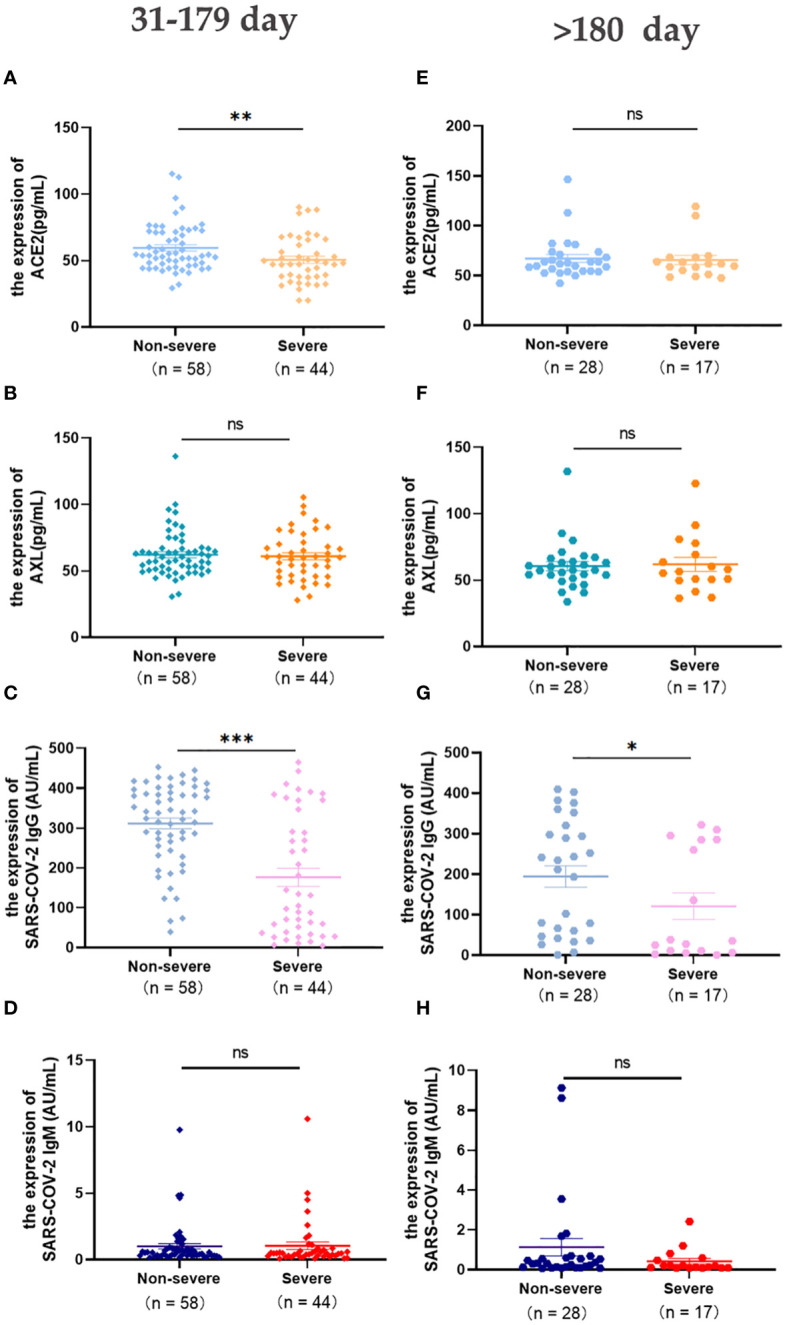
Expression of serum ACE2, AXL and SARS-COV-2 IgG/IgM in severe and non-severe groups at intervals of 31-179 days and >180 days were compared with Mann Whitney test **(A-H)** (*P <0.05; **P <0.01; ***P <0.001; ns, not statistically significant).

The change in these indicators were concerned within a half year and more than 180 days after laboratory-confirmed COVID-19. The levels of serum ACE2 and SARS-COV-2 IgG in the severe group were significantly lower than those in the non-severe group (Days 31-179) (**Figures 3A, C**). After 6 months, SARS-COV-2 IgG was still higher in the non-severe group than in the severe group (**Figure 3G**), and there were no significant differences in the other three indicators between the two groups(>180 days) (**Figures 3E-F, H**).

### 3.3 Evolution of serum ACE2, AXL and SARS-COV-2 IgG/IgM levels over time

**Figure 4:**
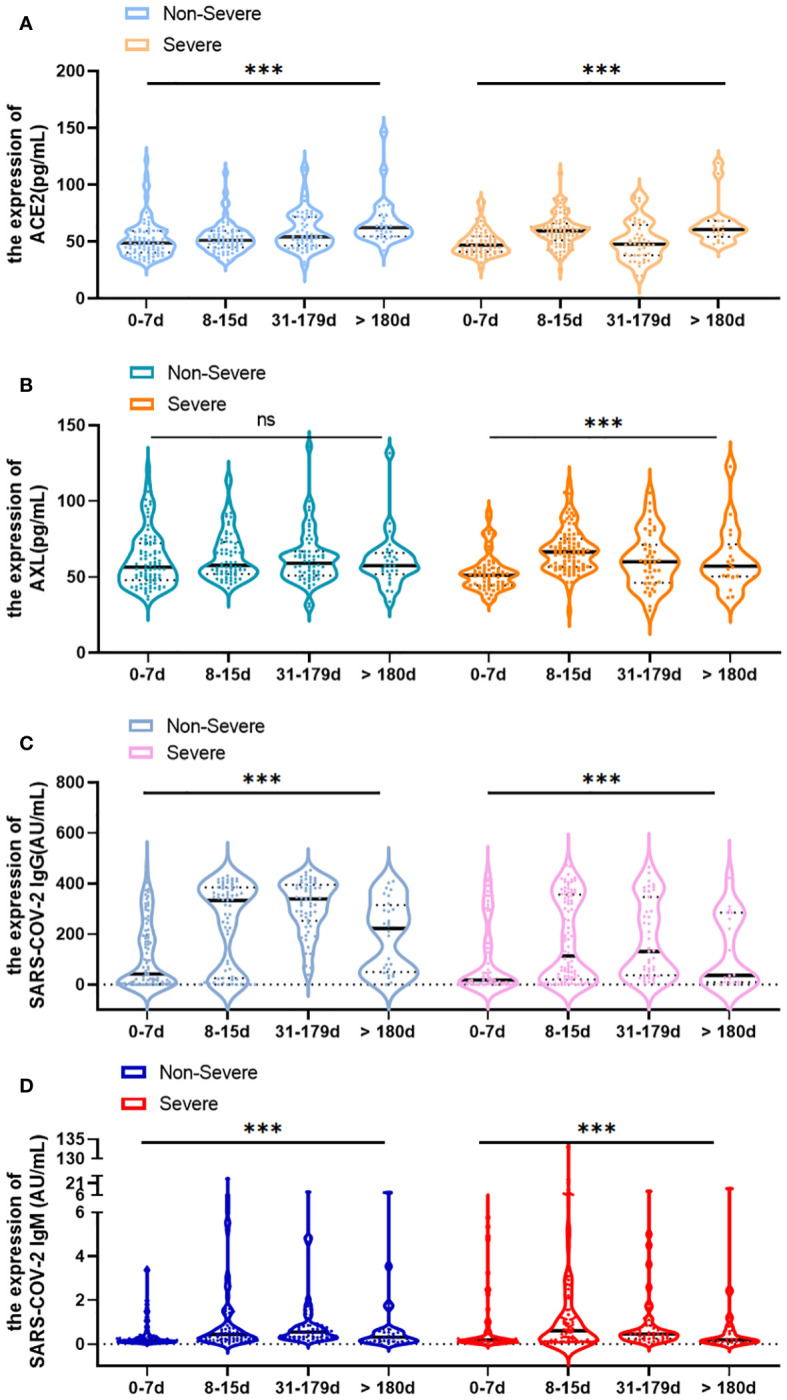
Change of serum ACE2, AXL and SARS-COV-2 IgG/IgM levels over time (0-7 days, 8-15 days, 31-179 days and >180 days) in severe and non-severegroups, respectively **(A–D)**. Data were presented as violin plots (median, quartiles and all points). The data do not follow normal distribution, statisticalsignificance of the difference was evaluated by Kruskal-Wallis test (***P <0.001; ns, not statistically significant).

The study illustrated the overall profile of serum ACE2, AXL, SARS-COV-2 IgG/IgM at different time points after infection. In the non-severe group, the level of ACE2 increased over time (**Figure 4A**). While the levels of SARS-COV-2 IgG and IgM were on the rise in the 0–179 days interval but declined after 180 days (**Figures 4C, D**). No statistically significant increase in the level of serum AXL was observed (**Figure 4B**). In the severe group, the serum levels of ACE2, AXL, and SARS-COV-2 IgM all reached a peak during 8–15 days, but subsequently decreased (**Figures 4A, B, D**). Moreover, the serum level of SARS-COV-2 IgG rapidly increased after infection but declined after 180 days (**Figure 4C**).

### 3.4 Changes of serum ACE2, AXL and SARS-COV-2 IgG/IgM by using paired Data

**Figure 5:**
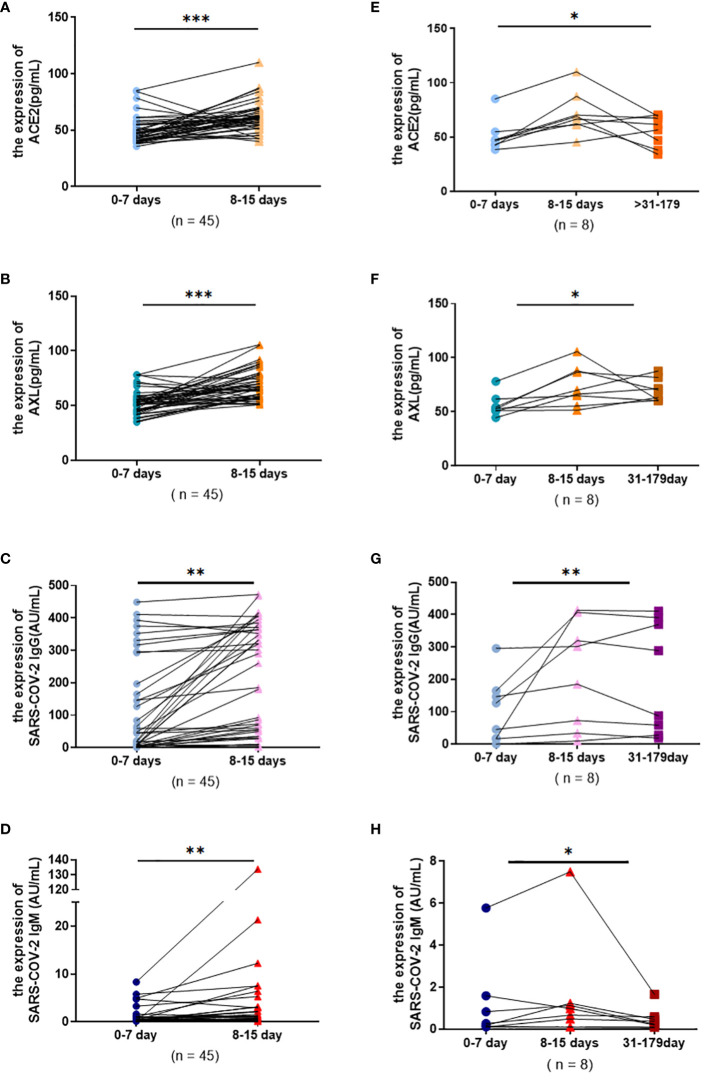
Serum ACE2, AXL and SARS-COV-2 IgG/IgM levels in severe group were compared among different temporal periods by using paired data. Wilcoxon matched-pairs signed rank tests were used to compare the changes of ACE2, AXL and SARS-COV-2 IgG, SARS-COV-2 IgM levels from 0-7 days and 8-15 days **(A-D)**. Friedman test were used to compare the changes of ACE2, AXL and SARS-COV-2 IgG, SARS-COV-2 IgM levels among 0-7 days, 8-15 days and 31-180 days **(E-H)** (*P <0.05; **P <0.01; ***P <0.001).

The paired data from severe patients with COVID-19 were conducted to investigate the change pattern of serum antibodies more precisely. The results showed that the levels of serum ACE2, AXL, SARS-COV-2 IgG and IgM all had a significant rise during 8-14 days (**Figures 5A-D**). In addition, the levels of ACE2, AXL, and SARS-COV-2 IgM were significantly decreased after 30 days, while SARS-COV-2 IgG was not obviously altered (**Figures 5E-H**).

### 3.5 Do the levels of ACE2, AXL and SARS-COV-2 IgG/IgM in COVID-19 patients return to an uninfected state after 180 days?

**Figure 6:**
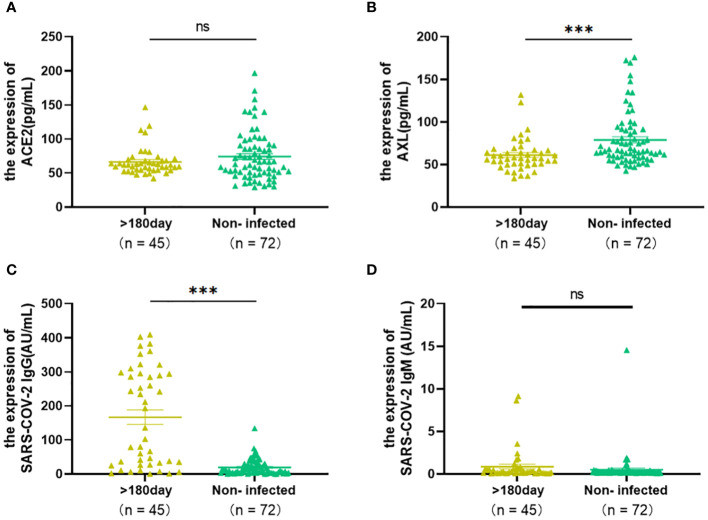
The Levels of ACE2, AXL and SARS-COV-2 IgG/IgM of COVID-19 patients (>180 days) were compared with those of uninfected individuals by using Mann Whitney test **(A–D)** (*P <0.05; **P <0.01; ***P <0.001; ns, not statistically significant).

To answer this question, the levels of serum ACE2, AXL, SARS-COV-2 IgG/IgM between 45 patients (>180 days) and 71 uninfected individuals were analyzed. The results showed that there were no significant differences in ACE2 and SARS-COV-2 IgM between the two groups (**Figures 6A, D**), indicating that the serum levels of ACE2 and SARS-COV-2 IgM returned to an uninfected stage. However, AXL was sustained at a lower level, and SARS-COV-2 IgG was still at a higher level in the infected group after 180 days compared with the uninfected group (**Figures 6B, C**).

## 4 Discussion

Based on the experience gained during the COVID-19 pandemic, new indicators need to be developed at an early stage to predict the emergence and severity of new pathogens, especially those associated with severe cases. Many studies have demonstrated that serum ACE2 activity correlated with COVID-19 severity and predicted mortality (13–15). In our study, not only ACE2, but also serum AXL, derived from another receptor of COVID-19, also seem to be a potential molecular marker for predicting COVID-19 progression.

Compared with the non-infected group, the initial cohort of COVID-19 patients showed with higher levels of SARS-COV-2 IgG/IgM. However, ACE2 in the COVID-19 group were significantly lower than those in the non-infected group, which is consistent with the result reported by María del (16). The level of AXL also decreased in the initial cohort of COVID-19 patients, which was first noted in our research. A previous comprehensive study on SARS-CoV had established that the binding of the S glycoprotein to ACE2 receptor down-regulated ACE2 expression (17). The SARS-COV-2 enters the cytoplasm through ACE2 and AXL-mediated endocytosis, which may cause the decrease in serum ACE2 and AXL levels in the initial cohort of COVID-19 patients. Surprisingly, the ROC curve analysis showed that the combined AUC of ACE2 and AXL did not exceed the AUC of AXL alone. Therefore, serum AXL seems to be a better predictor for COVID-19.

During the first week after infection with COVID-19, there is no obvious difference in symptoms and the levels of SARS-COV-2 IgG/IgM between non-severe and severe patients. However, better management can be achieved to prevent deaths by capturing early warning signs and timely intervention in critically ill patients. Fortunately, our results show that AXL, but not ACE2, can provide early warning of clinical deterioration. Then the changes of serum ACE2, AXL and SARS-COV-2 IgG/IgM between the non-severe and severe groups over time were observed. Serum ACE2 and AXL levels peaked at 8-15 days in the severe group compared to the non-severe group. Although the serum SARS-COV-2 IgG level in the severe group was always lower than in the non-severe group, it reached a significant peak at 31-179 days in both groups.

The main manifestations of SARS-CoV-2 infection were respiratory symptoms, but single-cell sequencing data showed that ACE2 expression was low in human tissues (18, 19). AXL is widely expressed in almost all human organs. In particular, in human pulmonary and bronchial epithelial tissue and cells, AXL expression is much higher than ACE2 expression (20). AXL is known to regulate various intracellular signaling pathways, including Ras/ERK, PI3K, and p38 (21, 22). SARS-CoV-2 activated the PI3K/Akt/mTOR signaling during the initial phases of infection (23, 24), which supported the theory of AXL as an early warning indicator for clinical deterioration. Another study found that the p38/MAPK signaling was promoted by SARS-CoV-2 infection leading to the overproduction of inflammatory cytokines (25). As is well known, SARS-CoV-2 triggers a strong immune response that can cause cytokine storm syndrome in severe COVID-19 patients (26). Hypoxia inducible factor-1 (HIF-1) binds to the AXL promoter, and then promotes SARS-CoV-2 infection and exacerbates inflammatory responses to COVID-19 (27, 28). Moreover, serum AXL has been reported to interact with two vitamin K-dependent protein ligands, growth arrest-specific factor 6 (GAS6) and protein S, forming a complex. Elevated levels of AXL complex have been detected in a variety of pathologies including specific inflammatory conditions and various tumors (29–33). Numerous studies have reported that plasma Gas6 levels are directly related to the severity and outcome of COVID-19 (34–36), and the Gas6/AXL axis is involved in regulating inflammation and fibrosis in COVID-19 patients (37). In our results, a significant change in serum AXL levels were found in the severe group but not in the non-severe group. Considering serum AXL plays a major role in regulating inflammation, AXL may play an important role in the severe progression of COVID-19.

After half a year, the serum levels of ACE2 and SARS-CoV-2 IgM returned to levels seen in uninfected individuals. Interestingly, AXL sustained lower levels, and SARS-CoV-2 IgG remained at higher levels in the infected group after 180 days. Some patients recovering from COVID-19 may develop a group of new onset or aggravated sequelae known as long COVID (38). The prevalence of long COVID is still uncertain, but evidence is emerging that it is relatively common (39, 40). There was a study indicated that ACE2 activity is substantially attenuated at 8 months post-infection and has not been associated with long COVID symptoms (41). Long COVID-19 can occur as part of the process of COVID-19. The major contributory mechanistic factor is the persistent cytokine storm that may last longer in long COVID patients than in others, probably triggered by aggregates of SARS-Co-2 discovered recently in the adrenal cortex, kidney and brain (42). AXL, playing an important role in regulating inflammation, which may correlated with the long COVID symptoms. However, further exploration and additional experimental validation are needed.

It should be pointed out that there were some shortcomings of this study. Firstly, we didn’t record the patient’s symptoms in detail in the telephone follow-up, so we can’t confirm whether the patients have post COVID-19 condition. Secondly, according to investigation and announcement of the Chinese Center for Disease Control and Prevention, the analysis of the novel coronavirus genome of COVID-19 cases in China showed that all were Omicron from 2022.9 to 2023.11, so all findings above apply only to Omicron infection.

Nevertheless, a study indicated that AXL could independently mediate the omicron infection (43). In our study, the level of serum AXL not only is correlated with the development of COVID-19, but also can provide early warning of clinical deterioration in the first week after infection, suggesting that it is a superior biomarker for COVID-19. Our results offer a new perspective for early management of COVID-19.

## 5 Conclusions

Previous study demonstrated serum ACE2 can be used as a protective biomarker for rapid test screening and even as one of the treatment strategies. In our study, AXL and ACE2 were compared in patient serum for the first time. Firstly, serum AXL appears to be a better predictor for COVID-19, with the highest AUC. Secondly, only AXL can provide early warning of clinical deterioration in the first week after infection with COVID-19. Thirdly, AXL and ACE2 play important roles in the severe progression of COVID-19. Lastly, AXL may be related to the development of long COVID symptoms. In summary, serum AXL seems to be a superior biomarker for COVID-19. However, further studies are needed to understand the immune mechanism involved.

## Data availability statement

The raw data supporting the conclusions of this article will be made available by the authors, without undue reservation.

## Ethics statement

The studies involving humans were approved by the Institutional Review Board of Fujian Provincial Hospital. The studies were conducted in accordance with the local legislation and institutional requirements. Written informed consent for participation in this study was provided by the participants’ legal guardians/next of kin.

## Author contributions

JY: Formal Analysis, Methodology, Software, Writing – original draft. RH: Software, Writing – original draft. RZ: Software, Writing – original draft, Data curation, Formal Analysis, Methodology. JS: Data curation, Writing – review & editing. SH: Formal Analysis, Methodology, Software, Writing – review & editing. YK: Formal Analysis, Software, Data curation, Writing – original draft. FC: Investigation, Validation, Writing – review & editing. JC: Methodology, Project administration, Visualization, Writing – review & editing. LC: Project administration, Conceptualization, Supervision, Writing – review & editing.
